# eHealth Promoting Stoma Self-care for People With an Elimination Ostomy: Focus Group Study

**DOI:** 10.2196/39826

**Published:** 2023-03-13

**Authors:** Igor Soares-Pinto, Ana Margarida Pinto Braga, Isabel Maria Ribeiro Morais Araujo Santos, Natália Maria Ribeiro Gomes Ferreira, Sandra Cristina da Rocha e Silva, Paulo Jorge Alves

**Affiliations:** 1 Instituto de Ciências da Saúde Universidade Católica Portuguesa Porto Portugal; 2 Escola Superior de Saúde Norte da Cruz Vermelha Portuguesa Oliveira de Azeméis Portugal; 3 Instituto Português de Oncologia de Coimbra Francisco Gentil Gabinete de Estomaterapia Coimbra Portugal; 4 Centro Hospitalar Póvoa de Varzim-Vila do Conde Gabinete de Estomaterapia Póvoa de Varzim-Vila do Conde Portugal; 5 Centro Hospitalar Universitário de Santo António Gabinete de Estomaterapia Porto Portugal; 6 Centro de Investigação Interdisciplinar em Saúde Porto Portugal

**Keywords:** self-care, ostomy, nurses, health education, telemedicine, eHealth

## Abstract

**Background:**

The construction of an elimination stoma has a physical, psychological, and social impact on the person. The development of stoma self-care competence contributes to the adaptation to a new health condition and improvement of quality of life. eHealth refers to everything associated with information and communication technology and health care, including telemedicine, mobile health, and health informatics. The use of eHealth platforms by the person with an ostomy, as a digital application that includes websites and mobile phone apps, can bring scientific knowledge and well-informed practices to individuals, families, and communities. It also allows functionalities that enable the person to describe and identify early signs and symptoms and precursors of complications and to be guided to an adequate health response for their problems.

**Objective:**

This study aimed to define the most relevant content and features to promote ostomy self-care integrated into an eHealth platform as a digital app or website to be used by patients for self-management of stoma care.

**Methods:**

We developed a descriptive, exploratory study with a qualitative approach using the focus group methodology, which was oriented to reach a consensus of at least 80%. A convenience sample of 7 participants consisting of stomatherapy nurses was used. The focus group discussion was recorded, and field notes were taken. The focus group meeting was fully transcribed, and a qualitative analysis was performed. The research question was: Which content and features for ostomy self-care promotion should be integrated into an eHealth platform as a digital app or website?

**Results:**

An eHealth platform, which can be a smartphone app or website, for people with ostomy should provide content aimed at promoting self-care, namely in the field of knowledge and self-monitoring, as well as the possibility of interacting with a stomatherapy care nurse.

**Conclusions:**

The stomatherapy nurse has a decisive role in promoting adaptation to life with a stoma, namely through the promotion of stoma self-care. Technological evolution has emerged as a useful tool to enhance nursing interventions and promote self-care competence. The development of an eHealth platform aimed at promoting ostomy self-care should include the capabilities for telehealth and help with decision-making regarding self-monitoring and seeking differentiated care.

## Introduction

### Background

An elimination ostomy is a surgically created opening in the abdominal wall that results in the diversion of feces or urine to the exterior; it may be permanent or temporary [1].

It is estimated that about 1 million people are living with an ostomy, and 100,000 to 130,000 new ostomies are created annually in the United States [2]. In Portugal, there is no clear evidence on the number of people with an elimination ostomy, but it is estimated that there are more than 16,000 ostomies [3]. It is expected that this number will increase since the most likely cause for its construction is colorectal or bladder cancer [4], incidences for which are expected to increase by 60% by 2040 [5].

In addition to the epidemiology, elimination ostomies can be classified according to the anatomical part involved and have different outcomes in patient quality of life and lifestyle. The presence of an ostomy is a life-changing event and has negative implications on various aspects of an individual’s quality of life. It affects those closest to the person, namely family members and caregivers [6]. The person has to deal with not only the diagnosis of a disease and its therapeutic implications but also physical, self-image, and emotional changes, which require necessary adaptations to daily life, including social and professional activities [7]. Therefore, training the person to take care of their ostomy is the responsibility of the clinical team, especially the stomatherapy nurse.

In Portugal, the stomatherapy nurse is a health care professional with advanced skills and knowledge in stoma care. Stomatherapy nurses have a fundamental role in the transition process. Their responsibility and competence to plan, define, implement, and evaluate interventions aim to adapt and modify the person’s reality and prevent complications, thus contributing to an effective training process to promote autonomy and self-care for the stoma [8].

Self-care represents the set of actions that the individual, as well as their family members, performs vis-a-vis their health and well-being, and these actions are directed to keep themselves in shape, maintain good physical and mental health, meet social and psychological needs, prevent illness or accidents, care for minor illnesses and long-term conditions, and maintain health and well-being after an acute illness or hospital discharge [6].

Easy access to information online emerges as a duality for risk and benefit, because if the patient is not able to assess the quality of the information and its suitability for their particular case, the decision-making process can have negative consequences [9]. Particularly in the context of a person with an ostomy, the benefit of this digital communication is providing scientific knowledge and information about well-informed practices to individuals, families, and communities. The person can also describe and identify early signs and symptoms and precursors of complications, as well as receive guidance on the adequate level of health response to their problems, contributing to easier, more appropriate decision-making and leading to reduced costs and economic impact on health [10].

In the scoping review carried out previously by the first author in the context of his doctoral thesis [11], literature was identified that addresses the use of digital tools to support people with an ostomy; however, the studies focused exclusively on communication between the person and the health professional [12].

In a qualitative study conducted in Portugal, also conducted by the first author, that analyzed the nurse's and patient's perspectives on the promotion of self-care for the stoma, nurses and patients referred to the use of the internet, email, videos, and images as resources. However, they did not refer to an internet site nor a specific tool that had all the resources needed [13]. In addition, we did not identify any platform that provides content aimed at promoting ostomy self-care.

These 2 studies made it possible to identify internationally available resources that promote stoma self-care and its limitations. On the other hand, the studies made it possible to identify the resources used in Portugal and the need to produce valid resources with clinical utility for the promotion of stoma self-care.

Someone undergoing the construction of an elimination stoma is required to have the knowledge and skills to autonomously and effectively manage their new condition. For this purpose, a specific, systematic intervention by nurses in the pre and postoperative periods and after discharge that is directed toward promoting stoma self-care positively influences the path of adaptation to the circumstance of living with an ostomy [14].

Self-care is thus an ongoing process, important for trust and involvement in the new health condition [6]. The development of stoma self-care competence improves the person’s results and is associated with a better quality of life, reducing readmission and complication rates [15].

In the current context of the digital age, the internet has emerged as a source of information, often used by patients and families to obtain information about diseases, treatment options, and care management; for some, it is their first source of information, even before consulting a health professional [16].

As the population interacts with digital technology in almost every aspect of their daily lives, they also expect faster access to answers about their health issues. This is why the intensive use of smartphone and mobile apps offers people new ways to self-assess and monitor symptoms [17].

The use of digital technology in health allows for increased availability of health information, giving people more access, options, and tools to access their health information and communicate with their health team [17].

However, digital literacy does not always match users' health literacy [9]; therefore, easy and quick access to digital health information can have some setbacks. The topics covered, as well as the quality of the content, can vary, and the authors and sources of this information are often unknown. The content can also range from a peer or professional review to personal blogs, opinions, or other people’s experiences [16]. The multiplicity of information can make it difficult and interfere with the selection of the most reliable information needed by the caregiver and the person with an ostomy in the management of their ostomy [18]. On the other hand, the perception of the quality of health information may change, and the target population may not have the health literacy necessary to assess health information and relate it to their specific condition or case [16]. In fact, it is not enough just to access information; it is also necessary to select, understand, and use it properly and for the intended goals of solving health problems [19].

The term electronic health (or eHealth) refers to health services and information delivered or enhanced through the internet and related technologies [20]; however, this term also can be assumed to be the broadest umbrella encompassing everything that comes with information and communication technology and health care, including telemedicine, mobile health, and health informatics [21], considering eHealth as a tool to promote health or improve health care [22].

In this context, the development of an eHealth platform for support immediately after hospital discharge has emerged; this time period is one of greater vulnerability and when stoma and peristomal skin complications occur. Thus, such an eHealth platform could contribute to reducing the incidence or severity of complications [23]. For this, it is necessary to understand the type of platform and functionalities that would benefit the person with an ostomy.

An eHealth platform may also facilitate the dissemination of guidelines and information regarding the care and self-care of the person with a stoma, strengthening communication as well as the family’s emotional aspects, positively contributing to the transition of the health-disease process [24].

### Objectives

The research question that guided this study was the following: Which content and features should be integrated into an eHealth platform as a digital app or website to promote ostomy self-care? Thus, the aim of this study was to contribute to the development of an eHealth platform and define the content and features to be included in this type of platform to promote self-care of the person with an elimination ostomy.

## Methods

This was a descriptive, exploratory study with a qualitative approach. We complied with the consolidated criteria for reporting qualitative research (COREQ) with the aim of promoting explicit and comprehensive reporting of interviews and focus groups [25]. The focus group was conducted according to the methodological guidelines defined by Krueger and Casey [26].

### Recruitment

A total of 7 participants were involved in the focus group. The number of participants must be sufficient to create discussion, as too large a group may prevent some participants from sharing their ideas within the time available. In this sense, the group size should be from 4 to 12 participants: “the ideal size of a focus group for most noncommercial topics is five to eight participants” [27,28].

The inclusion criterion for the participants was that they had to have advanced competence in stomatherapy as defined by the Portuguese Nursing Board [29].

The sampling process was intentional, seeking to obtain balance in the participants, in order to have professionals with experience in all contexts of care for people for whom construction of an ostomy has been proposed, including the preoperative intervention during the consultation, care of the postoperative inpatient, and follow-up consultation after discharge.

All participants approached agreed to participate in the study.

In order to obtain maximum variation in the participants' experiences, differences were considered related to the institutional dynamics and location of the institution, whether in urban or rural areas.

The participants were contacted by email through the Portuguese Association of Stomatherapy Care Nurses (APECE).

In the invitation addressed to the APECE, the motivation for the investigation, its relevance, and the promotion of self-care as a central topic of discussion were explained. No one declined the invitation nor withdrew participation during the study.

### Ethical Considerations

Approval was obtained from the ethics committee for health of the Universidade Católica Portuguesa to develop the eHealth platform to promote self-care for people with an elimination stoma (number 141, 246). The confidentiality of all participants was guaranteed, and they were informed that they could withdraw from the study at any time. All participants gave their formal written consent. The location selected for the meetings was at the center of Portugal to facilitate the movement of participants from various parts of the country.

### Procedure

Regarding the realization of the focus group, having already had experience in this methodology, the main researcher led the discussion group. The objective was explained, and the exchange of ideas was encouraged, with a second member recording the proceeding and observing the group. This focus group met twice, for approximately 2 hours each session. A script was constructed based on a scoping review on nursing interventions to promote self-care in people with an elimination ostomy [11]. The script also considered the domains that make up the competence of self-care: knowledge, self-surveillance, interpretation, decision-making, execution, and resource management [30]. This script, which was used to conduct the meetings, was based on the questions listed in Table 1.

The literature review carried out and already published [11] was conducted by the first and last authors, being an integral part of the first author's PhD in nursing.

To ensure balance and the achievement of expected results, the group discussion was led by the principal researcher, who ensured equality among group members in terms of intervention in decision-making, while ensuring that all ideas were carefully heard and considered.

Regarding the research assumptions, a consensus of 80% was defined from the outset [31] for all content, asking participants if they agreed with the information and method of exposition, assuming that, if 80% of consensus was not reached, interactive dialogue would continue until 80% agreement was reached.

We also ensured that all issues would be examined in detail and dissenting opinions were duly considered.

Analysis of the information was conducted immediately after the session; no software was used. However, for further analysis, field notes were made throughout the session, to guarantee the consideration and possibility of confirmation that all data were taken into account.

For the data analysis in the first phase, the first 2 authors viewed the recording of the meeting and compared it with the field notes to add aspects not identified in the recording. In the second phase, after transforming the transcribed content into raw data, prepared for grouping, the coding phase was carried out. Categorization followed, which involved the organization and classification of selected text and key points of the transcribed discussions, in context units to form codes. Finally, the interpretation process was concluded, which involved the inferential process that represented the explanation of the codes of the emerging categories and subcategories. Two authors (ISP and AMPB) coded and validated the categories, with approval being obtained from the remaining authors.

The researchers' pre-understanding guided the analysis. All authors held critical discussions, questioning their pre-understanding and theoretical knowledge, to reduce investigator bias throughout the research process.

The authors, who are stoma care nurses, required constant awareness of the need to reduce the risk of portraying their professional experiences and perceptions during all stages of the study.

This pre-understanding was reflected, reconsidered, and examined by the investigators during the process of analyzing and interpreting the data.

To ensure valid and grounded interpretations of the data, we sought to maintain a critical and honest posture through self-reflection.

In the data analysis, we tried to align the theory, objective of the study, data collection, analysis, and results.

**Table 1:** The focus group’s guiding questions.

Question number	Question
1	What is the benefit of using an eHealth platform to promote self-care for an elimination ostomy?
2	What are the central aspects related to promoting self-care for people with an elimination ostomy that should be considered in constructing an eHealth platform?
3	What content related to promoting stoma self-care should be part of an eHealth platform intended to promote self-care for bowel elimination stoma?
4	What strategies can be used to present content on an eHealth platform to promote self-care for the bowel elimination stoma?
5	In the context of promoting self-care for the bowel elimination stoma, what features should an eHealth platform contain?

## Results

The focus group consisted of 7 participants, mostly women, from various parts of the country: 2 from the north, 3 from the center, and 2 from the south of Portugal. We provide their main characteristics in Table 2.

The focus group was formed by the board members of the APECE, an association of recognized relevance in the field of stomatherapy in Portugal.

Regarding the benefits of using an eHealth platform, there was consensus that it responds to an extremely current need, related to the need for remote monitoring. The greatest difficulty in interacting with users and using technologies as support tools in the health area is related to the recognition that there may be population groups with lower possibility of accessing this tool, namely older people.

The results obtained with the focus group were divided into 3 themes: (1) central aspects of self-care, (2) content and methods for promoting self-care, and (3) features of an eHealth platform to promote self-care for bowel elimination stoma.

**Table 2:** Characteristics of the focus group participants (n=7).

Characteristics of the participants			Results
**Gender, n (%)**			
	Male	1 (14)	
	Female	6 (86)	
Age (years), mean (range)			45.6 (31-59)
**Length of experience (years), mean (range)**			
	Nurse	22 (8-33)	
	Ostomy care	19 (2-28)	
	Stomatherapy consultant	12 (2-20)	
**Nurse specialty, n (%)**			
	Rehabilitation nursing	1 (14)	
	Medical-surgical nursing	4 (57)	
	Mental health and psychiatric nursing	1 (14)	
	Community nursing	1 (14)	
Had experience with teaching nursing, n (%)			7 (100)
Postgraduate studies in stomatherapy, n (%)			7 (100)

### Central Aspects of Self-care for Bowel Elimination Stoma

Of the domains that make up the competence for stoma self-care, the possible aspects to integrate into an eHealth platform were discussed.

Knowledge and self-surveillance were unanimously considered. An expert said that interpretation and decision-making can also be areas enhanced by the platform, namely identifying complications and the decision to resolve them or seek differentiated help from a stomatherapy nurse:

A patient looking at the image and comparing it with their stoma can recognize the difference and seek help...

Nurse 1

### Contents and Methods in Promoting Self-care for the Bowel Elimination Stoma

In the focus group discussion focused on nursing interventions, we tried to define the content and method of promotion to be used in the eHealth platform, as described in Table 3. The content and methods were obtained by consensus greater than 80% (≥6 experts).

It should be noted that issues emerged that, despite not having reached consensus ≥80%, were representative, namely the dietary regimen, with 5 participants suggesting it should be integrated:

Food is essential for the integration of this new condition into their daily lives.

Nurse 3

However, it was concluded that the type of stoma, underlying pathology for the stoma construction, possible neoadjuvant treatments, and person’s associated comorbidities are highly variable factors, which is why it is too complex to establish recommendations in terms of a feeding pattern that considers all the variables listed.

Two experts suggested that sexuality be included:

The approach to sexuality could be facilitated by the use of this tool...

Nurse 5

However, the participants discussed that sexuality could be included, but only some general aspects and information directed toward the available resources should be included.

The group concluded that both diet and sexuality are very relevant topics, but they are not directed toward stoma self-care; they are part of other self-care areas with an impact on integrating this new condition. However, their importance was recognized for the integration of the new condition and promoting quality of life.

**Table 3:** Content and methods for promoting self-care for the bowel elimination stoma to be integrated into an eHealth platform (n=7).

Content			Method		Consensus, n (%)
1. Elimination ostomy: definition, anatomy and physiology, digestive system, urinary system, types of elimination ostomies, colostomy, ileostomy, urostomy, nephrostomy, collecting devices			Images and text		7 (100)
2. Pouching system: according to ostomy type, number of pieces, type of fixation (adhesive and mechanical)			Images and text		7 (100)
3. Stoma site marking			Images and text		6 (8)
4. Stoma self-care: hygiene, trichotomy/hair removal, device removal, device/plate clipping, device application (single piece and 2 pieces)			Text and video		7 (100)
5. Stoma and skin self-surveillance					
	Stoma observation: standard for normality, stoma complications (stenosis/squeezing, prolapse, ulcer, hemorrhage/blood loss, retraction)	Real images and text		6 (85)	
	Skin observation: standard for normality, skin complications (maceration, erythema, hernia, crystal deposits [oxalate/phosphate] on the skin, wounds [ulcer, pressure ulcer, excoriation])	Real images and text		6 (85)	
6. Dietary regimen: colostomy, ileostomy, urostomy			—^a^		5 (71)
7. Sexuality			—		5 (71)
8. Health resources: stomatherapy consultation, pouching system and accessories reimbursement, pouching system and accessories distribution, consume limit on pouching systems and accessories			Text		6 (85)

^a^Did not reach at least 80% consensus, so methods were not discussed.

### Features of an eHealth Platform to Promote Stoma Self-care

Regarding a set of priority features inherent to an eHealth platform, the features listed in Table 4 were understood as essential in a digital tool with the objective of promoting self-care competence for the person with an elimination ostomy.

The possibility of requesting samples of ostomy devices and accessories from the industry through the platform was also discussed; however, there was no consensus from the working group, considering that all materials must be subject to a prior and joint evaluation by the stomatherapy nurse and the person with an ostomy. Before its introduction:

it is a very great risk to give the person the possibility to try devices and accessories, without a previous evaluation by the stomatherapy care nurse...

Nurse 7

The possibility for the platform to include a space for an information sharing forum was also explored. It was understood, however, that due to the difficulty in screening the information transmitted, this item should not be included due to the requirement for a moderator:

If there is not a very strict control, less suitable content can be placed that can be read and misused...

Nurse 6

**Table 4:** Functions to be integrated in an eHealth platform aimed at promoting stoma self-care.

Features	Characteristics
Telehealth	Interaction via text message, images, or videos with a stomatherapy nurse: The purpose is not to replace face-to-face contact by the health care team but to add an extra resource in case of doubts about or to promote self-care.
Self-surveillance	Self-surveillance algorithms for the stoma and peristomal skin: The purpose is to help patients identify changes and solutions to problems with the stoma and skin.
Information about self-care and health resources	Information in the form of videos, images, and text directed at promoting stoma self-care: Make relevant normative and legislative documents available for the person with an ostomy. The purpose is to provide relevant and reliable content and help the patient identify available resources and services.

## Discussion

### Topics With Consensus

The results show that consensus was reached regarding eHealth tools as current aids with numerous advantages in the context of managing the condition of the person with a stoma. This is corroborated by the literature, which shows the added value of eHealth tools in disease management and health promotion, as well as the enormous potential to promote patient engagement [32].

Considering the 6 domains that integrate the competence of self-care, knowledge, self-surveillance, interpretation, decision-making, execution, and management of health resources [30], a digital platform focused on promoting self-care can contribute to the development of knowledge and self-surveillance domains. In addition to these domains, which are also supported by evidence [33], eHealth platforms have also demonstrated impact at the behavioral level, namely by the promotion of skill development and disease self-management [34].

In fact, acquiring knowledge and skills will contribute to the promotion of self-surveillance. Several studies have pointed out that the person with more knowledge about their condition or disease can more easily promote stoma self-care [35].

With regard to the content and methods to be included in an eHealth platform, the group of experts suggested 8 themes to be integrated into the domain of knowledge. Only 6 reached consensus superior to 80%, and the sexuality and dietary themes reached 71% consensus.

Regarding the proposed content of “elimination ostomy” (content 1 in Table 3) and “pouching system” (content 2 in Table 3), the person or caregiver must understand the anatomical aspects to facilitate the understanding of the type of stoma that was constructed, as well as its function and effluent management. These are basic aspects for the promotion of awareness, an aspect that is necessary for promotion of self-care [36]. This content is useful as a starting point for training for self-care, and the availability of this information, even in the initial stages, will contribute to knowledge of the condition. In addition, in later stages, it can be integrated into the decision process, leading to greater involvement in decision-making regarding the devices to be used.

“Stoma site marking” (content 3 in Table 3) is associated with improved quality of life, reduced complications, and better adaptation to the new condition [14]. Marking the stoma site presupposes having a set of assumptions as a basis to identify the best site for its location. These assumptions, in addition to the visible characteristics, which include skin folds, bony prominences, and abdominal morphological aspects, also include the person’s daily activities and clothing [37]. In this way, prior knowledge of these assumptions, the therapeutic route, and its purpose will allow the person to be involved in the decision-making process, facilitate the procedure for marking the stoma site, and reduce preoperative anxiety [1].

To promote “stoma self-care” (content 4 in Table 3), the person needs to receive adequate information as well as support to acquire the skills and resources needed to practice self-care [38].

A person with a stoma is required to have a set of knowledge and skills and the ability to integrate these into the management of stoma care and activities of daily living. The person with an elimination stoma must understand the type of stoma and whether it is temporary or permanent; know the appropriate material to use; know of alternative materials in case the equipment proves to be difficult to use or causes skin problems; have the ability to solve stoma-related problems, including recognizing changes in the stoma, characteristics of elimination, and peristoma skin; and know who to contact if any complications arise [39,40].

Content 5 (Table 3), “stoma and skin self-surveillance,” is also crucial to prevent complications, since approximately 80% of people with an ostomy will experience at least one complication during their lifetime [15] and the absence of complications favorably contributes to the adaptation and integration of the person’s new condition [41].

The person with an ostomy must, therefore, be aware of the appearance of changes and seek help that is appropriate to their needs. People with ostomies accompanied by stoma care nurses require 70% less care time, have fewer hospital readmissions due to complications, have one-half the average direct cost of treating complications, and report higher levels of well-being and quality of life and less pain [7].

The use of “health resources” (content 8 in Table 3) to improve the educational process and follow-up of patients through information and communication technologies is rapidly evolving, and nurses play a central and privileged role in the use of these technologies to improve and optimize their interventions with patients [42].

The creation of the stoma involves not only the need for a device but also a new body image that needs to be reconstructed, which is why it is a process that is simultaneously subjective and deeply reflective and requires careful intervention [43].

### Topics Without Consensus

“Dietary regimen” (content 6 in Table 3) is a very comprehensive topic and specific to each condition, especially for the person with a bowel elimination ostomy.

People with a colostomy have all their small intestines and some functioning colon. The risk of dehydration and malnutrition is therefore not significantly increased. On the other hand, people with an ileostomy do not have a functioning colon and will have varying lengths of functioning small intestine above the ileostomy [44].

Dietary advice is considered an important component of stoma management and is provided by many health care professionals, such as stoma nurses, dietitians, surgeons, gastroenterologists, and other specialist nurses [45].

The literature is not clear about the implications of different stomas and the length of functional intestine on dietary management [45]. Considering that providing recommendations about diet in an eHealth platform is complex, however, this can be a relevant topic to explore and define key points for different types of stomas.

“Sexuality” (content 7 in Table 3) is a highly individualized area of ​​a person’s life and related to all the conditions involved in the health and disease process. Living with an ostomy can have a more noticeable negative impact on aspects of body image and sexuality [1]. If, on the one hand, the person with an ostomy expresses several physiological changes in terms of sexuality, on the other hand, they may also experience psychological problems, such as fear and anxiety related to sexual performance and the possibility of accidents with the device during intimacy. Although the group of researchers considered this an important and integrative item in following up the person with an ostomy, its approach requires an individualized intervention guided by a health professional who knows methodologies and tools, such as strategies aimed at approaching sensitive topics such as sexuality [46]. Thus, in view of this and considering the unilateral approach of an eHealth platform, its inclusion may have negative repercussions on the intervention process and can only be considered the first phase of addressing the person's sexual problems—permission, with information directed at the availability of the stomatherapy nurse to address this issue and find solutions together [46].

### Features of an eHealth Platform

Regarding the features of the eHealth platform, rapid advances in technology and internet access have become not only a viable way to carry out educational interventions but also a platform that can be widely disseminated and implemented. In addition, internet interventions and programs that can be disseminated through the internet allow program content to be standardized, targeted to specific ages and developmental stages, and easily updated [47].

Faced with the challenge of an ostomy, the task of empowering the person with an ostomy is up to health professionals, namely stomatherapy nurses, providing them with knowledge and skills to manage their self-care. This task is usually carried out face to face in a programmed teaching context; however, the use of digital technologies favors more flexible access to information, allowing the person to search for and process it at their own pace [48].

The use of telehealth after hospital discharge is effective in improving satisfaction with care, reducing stoma complications, improving self-care competence, and increasing the patient’s self-confidence in dealing with the ostomy. Although performed at a distance, follow-up has become an extremely important factor for better adaptation to the ostomy [49]. There are, however, barriers to the use of digital information, which include lack of access to the internet and a low level of digital literacy in health [17].

With regard to the use of surveillance algorithms for the stoma and peristomal skin, continuous monitoring is one of the several areas of intervention by nurses that contribute to managing the new health condition of the person with an ostomy [1].

### Limitations

Despite the relevance of the results obtained, this study has some limitations. The discussions were held only once in the group, although 2 meetings were held, and whether the participants’ perception remained constant regarding the final results was not evaluated.

The literature review carried out and directed at nursing interventions to promote self-care was shared with the participants only on the day of the meeting, with no time for discussion of the information and confrontation with each person’s experience and taking the limitations of their own review into account [11].

Obtaining consensus has inherent limitations, namely not generating new knowledge but reflecting the opinion of experts. The results obtained could be different in larger groups.

### Conclusion

People with an elimination ostomy have their perspective of life altered, due to fear and doubts about the ostomy and devices to be used. Furthermore, people must manage various changes that occur in their daily lives, namely eating habits, hygiene, physical activity, and professional activity, as well as many other aspects necessary for their adaptation, which will have implications on their lifestyles [41,50].

Regardless of the approach, face-to-face or through eHealth platforms, the stomatherapy nurse has a wide field of action, interventions, and strategies to care for people with an ostomy and can enhance the person’s adaptation to their new condition.

This study made it possible to define a set of content and key features to be included in an eHealth platform focused on promoting self-care.

It is necessary to evaluate these new ways of communicating with patients with an ostomy and caregivers, which can facilitate the promotion of self-care and autonomy and can enhance social reintegration, through specialized support and monitoring.

Despite so many challenges and the complexity of the situation, when the person with an ostomy is adapted with adequate support and resources, it is possible to live an active and quality life [51].

## Abbreviations

APECE: Portuguese Association of Stomatherapy Care Nurses
COREQ: consolidated criteria for reporting qualitative research
